# Women's mental health in Mozambique: is maternity a protective factor?

**DOI:** 10.1017/gmh.2022.1

**Published:** 2022-02-11

**Authors:** Saida Khan, Pamela Scorza, Kathryn L. Lovero, Palmira dos Santos, Wilza Fumo, Barbara Camara, Maria A. Oquendo, Milton L. Wainberg, Marcelo Fejo, Cristiane S. Duarte

**Affiliations:** 1 Department of Mental Health, Ministry of Health, Av. Eduardo Mondlane/Av. Salvador Allende P.O. Box 1613, Maputo, Mozambique; 2 Department of Psychiatry, University of Pennsylvania Perelman School of Medicine, 3535 Market Street Suite 200, Philadelphia, Pennsylvania, USA; 3 Department of Psychiatry, New York State Psychiatric Institute and Columbia University Vagelos College of Physicians and Surgeons, 1051 Riverside Dr. Unit #24, New York, New York, USA; 4 Department of Psychiatry, Universidade Federal de São Paulo, Rua Major Marrigliano, 241, São Paulo, São Paulo, Brazil

**Keywords:** Mental Health, women, childbearing age, Southern Africa, Mozambique

## Abstract

**Backgroud:**

Globally, women have been shown to have high rates of common mental disorders (CMDs). In low and middle-income countries (LMICs), women face significant challenges related to maternity. However, no study has compared mental health problems among pregnant/post-partum women, childless women of childbearing age, and women with children in a low-income country. We sought to compare the frequency of CMD and suicide risk in a sample of women presenting or accompanying patients in primary care in two Mozambican semi-urban settings.

**Methods:**

We administered the MINI International Neuropsychiatric Interview to 853 women, of whom 220 (25.8%) were pregnant/post-partum, 177 (20.8%) were non-pregnant and childless, and 456 (53.5%) were non-pregnant and with children more than 1-year-old. Logistic regression models compared the likelihood of a psychiatric disorder across groups, adjusting for sociodemographic and chronic-illness covariates.

**Results:**

We found a high frequency of CMD and suicide risk among all women in this low-income context sample. In adjusted models, no differences in rates of depression, anxiety, or panic disorder were observed among groups. However, suicide risk was higher in women without children than pregnant/post-partum women.

**Conclusion:**

The frequency of CMD among women of childbearing age in our study was higher than documented rates in high-income countries and other LMIC. Additionally, we found that motherhood was not protective and that pregnancy and the postpartum period were not stages of increased risk for most disorders. This highlights the need to expand mental health services not only for perinatal women but all women of childbearing age in this and possibly similar settings.

## Introduction

It has been well-described around the world that women have higher rates of common mental disorders (CMDs), particularly anxiety and depression, compared to men (National Institute for Health and Care Excellence: Clinical Guidelines, 2009). Rates of CMDs are high around the childbearing years, with average rates of antepartum depression reaching 7–15% in high-income countries and 15–25% in low-and middle-income countries LMICs (Fisher *et al*., 2012; Gelaye *et al*., 2016; Woody *et al*., 2017), The prevalence of postpartum depression varies from 10% in high-income countries to approximately 20% in LMICs (Gelaye *et al*., 2016). Compared with the age-matched women in the general population, women in their second and third trimesters of pregnancy appear twice as likely to have clinically significant depressive symptoms (Bennett *et al*., 2004). *Prevalence studies* estimate that the rate of perinatal depression among women in the African region ranges from 11.3% to 18.3%, and a study reported that 14.8% of women experienced prenatal anxiety and 14% had postnatal anxiety (Sawyer *et al*., 2010)

In low and middle-income countries (LMICs), childbearing comes with significant risks and challenges, including poverty, high infant and maternal mortality rates, overcrowding, poor sanitation, malnutrition, malaria, HIV, and a lack of appropriate medical services (*Global report on urban health: equitable, healthier cities for sustainable development*, 2016). In such contexts, perinatal mental disorders are particularly likely to cause suffering, not only for the affected woman but also for her family and the health and development of her children (Miranda and Patel, 2005; National Institute for Health and Care Excellence: Clinical Guidelines, 2009).

Therefore, perinatal mental health has been the focus of much recent research in LMICs (Williams, 2018; Macginty *et al*., 2020). However, being a childless woman during childbearing years might be associated with different probabilities of having a mental disorder depending on the relative importance of childbearing for a woman's identity in a given socio-cultural context (Hollos and Larsen, 2008; Lisboa and Letras, 2016). In most cultures, having children is viewed as necessary to a woman's life to some degree. In more traditional societies, however, it is often considered fundamental. Children play a crucial role in cultures whose economies rely primarily on subsistence agriculture, providing labor in the fields and caring for younger children (Dyer, 2007; Hollos and Larsen, 2008; Lisboa and Letras, 2016). Perhaps relatedly, large families bring status, and a wife is considered successful if she can bear children. In such contexts, infertility is often highly stigmatized, is associated with intimate partner violence, and is often grounds for divorce or taking another wife (Hollos and Larsen, 2008; Lisboa and Letras, 2016).

In Mozambique, a low-income country in southeastern Africa, childbearing is considered paramount to a woman's identity. The failure to have children within marriage is highly problematic for a woman (Lisboa and Letras, 2016; Pinto, 2017). In addition to the traditional expectations of a woman's role in regards to childbearing, Mozambique has a high rate of HIV/AIDSs, with 13.2% of the population affected and up to 24.4% in some regions of the country, and women are more likely to be HIV positive (Ministério Da Saúde- Misau and Instituto Nacional De Estatística – Ine, 2015). Failure to bear children in this context could also be associated with the suspicion that a woman may have HIV given its risks in childbearing. This perception could cause added stigma and social exclusion for childless women (Ministério Da Saúde- Misau and Instituto Nacional De Estatística – Ine, 2015).

The treatment gaps – discrepancy between individuals needing treatment and those receiving treatment – are wide even in economically developed countries. The failure to detect and provide treatment to women suffering from CMDs may contribute to risk for poor health outcomes, suicide, alcohol and other substance abuse, and HIV infection from mother to child (Collins *et al*., 2006; Betancourt *et al*., 2010). More than 90% of the population in Mozambique do not have access to adequate health care (Dos Anjos Luis and Cabral, 2016), and mental health care is minimal, with fewer than 500 mental health providers nationwide (Dos Santos *et al*., 2016). However, the government of Mozambique has recognized the need for mental health treatment. Local officials are working with international partners to implement mental health treatment in the community and primary care (Wainberg *et al*., 2020, 2021).

While numerous studies in LMIC have highlighted the need for mental health services for women in the perinatal period (or pregnant and postpartum women), we have been unable to find one which examined this need relative to childless women, women with children more than 1-year-old and of child-bearing age. We seek to determine which of these groups of women have a higher are at higher risk for CMD as well as their suicide risk in a convenience sample of women presenting or accompanying patients in primary care in a semi-urban setting in Mozambique. We hypothesize that women in the prenatal and postpartum periods are at the most significant risk of having CMDs, followed by childless women of childbearing age and finally women with children older than 1 year. Our results will be used to direct the scale-up of services in Mozambique and may guide further research in similar settings, where the mental health needs of women of childbearing age may differ from those of women in higher-income contexts.

## Methods

### Study setting and subjects

We conducted a cross-sectional study in two provinces in Mozambique in 2018. Maputo is the capital city of Mozambique, located in the southern part of the country, with relatively higher socioeconomic conditions. Nampula is located in the northern part of the country, primarily rural, with socioeconomic conditions similar to nationwide estimates (Maunze *et al*., 2019). In Maputo, data were collected at two primary health care clinics and one district-level hospital that provides primary care, emergency, and outpatient mental health services. The hospital also includes in-patient health and psychiatric services as well as care for victims of interpersonal violence. In Nampula, data were collected at three primary care clinics that provide primary care and emergency services but do not have in-patient or outpatient mental health services.

### Study population

Participants included in this study were the subset of female participants of childbearing age (18–49 years) from a more extensive investigation to validate mental health screening tools in Mozambique in all patients at entry points at those facilities. At the beginning of each data collection day, male and female patients and their companions (18 years old or above) in waiting rooms at the health facilities watched a mental health presentation. After, they were invited to participate in this study. In total, 937 women accepted the invitation. They were taken to a private area, where research assistants presented the study overview, including objectives, eligibility and procedures and, if eligible, asked participants to provide written informed consent. Potential participants were excluded if they were minors (under 18) and/or could not communicate sufficiently in Portuguese, which interviewers assessed by asking potential participants to repeat the study objectives in their own words. Of the 937 women, 66 (7.0%) were excluded for not being of childbearing age; 871 (93.0%) were of childbearing age and met the criteria to participate in the study.

### Data collection

Data were collected through face-to-face interviews conducted by trained research assistants (Mozambican psychologists and psychology students in their final year of training). All questionnaires and interview responses were recorded on tablets using the REDCap data collection platform (Harris *et al*., 2009) a metadata-driven methodology and workflow process for providing translational research informatics support, hosted at the Foundation for Professional Development in Pretoria.

Assessment of CMD diagnoses (major depressive episode, generalized anxiety disorder, panic disorder) and moderate/high suicide risk (including suicidal thoughts, intent, and/or attempts) were made using the Mini International Neuropsychiatric Interview (MINI) (Sheehan *et al*., 1998). The MINI is a brief, structured interview that has been used as a diagnostic reference across many contexts (Ali *et al*., 2016). For the present study, we made minor local language adjustments to a Portuguese version that has been validated in the Brazilian population (De Azevedo Marques and Zuardi, 2008). The revised MINI was then locally tested through cognitive interviews (Willis, 2004) in a pilot with ten Mozambican adults at two health facilities. Data on self-reported socio-demographics (age, gender, marital status, living situation, education, religion, monthly household income, occupation, and ethnicity) and health (presence of chronic physical disorders, history of mental illness diagnosis and treatment, pregnancy, and parity) were collected through a demographic questionnaire. Only 12 /871 (1.4%) participants reported a history of mental illness diagnosis, likely owing to extreme under-identification of mental health problems in this low-resource setting. We had a risk management protocol in place for women reporting suicide risk or other common mental health disorders. Researchers referred these individuals to treatment in the Sanitary Unit, where they were offered treatment from health professionals.

### Data analysis

We assessed differences in the diagnosis of CMDs, comparing three groups: (G1) women who were pregnant or had a child under 1 year of age, (G2) women who never had children, and (G3) women with children above 1 year of age. ANOVA or *χ*^2^ tests were used for descriptive univariate comparative analysis, including age, marital status, employment status, family composition, living situation, level of education, and HIV status. Multiple logistic regression models were used to determine if the three groups differed concerning the different types of psychiatric disorders examined, adjusting for sociodemographic and clinical characteristics.

## Results

**Fig. 1.**
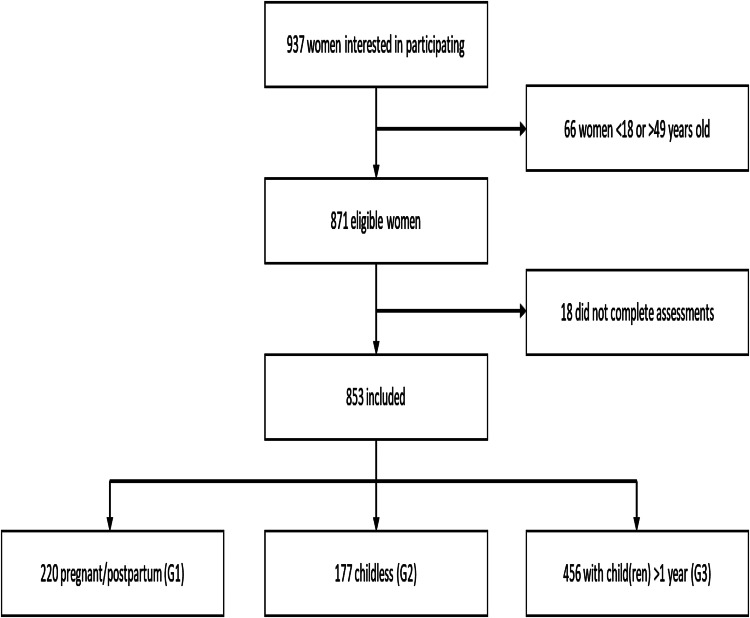
Profile of studied sample.

Figure 1 shows the profile of the studied sample. Of the 871 women of childbearing age, 18 did not complete the MINI interview. Of the remaining 853 women, 220 (25.8%) were pregnant or postpartum women (G1),

177 (20.8%) were non-pregnant women without children (G2), and 456 (53.5%) were women with children more than one-year-old (G3). The mean ages of mothers in G1, G2, and G3 were 25.9 years, 25.4 years, and 32.1 years, respectively (Table 1). Table 1 highlights the significant associations between various sociodemographic characteristics and maternity status. Age, marital status, living situation, occupation, HIV status, and presence of chronic illnesses were significantly different among maternity categories. In the subsequent logistic regression models, we adjusted for demographic characteristics in Model 2 – age, marital status, and occupation (the living situation was highly correlated with marital status). In Model 3 we additionally adjusted for health characteristics – HIV status and presence of chronic illnesses.

**Table 1.** Sociodemographic characteristics by motherhood category (*N* = 853)

	Pregnant or Postpartum (G1) *n* = 220	Childless (G2) *n* = 177	Child(ren) over 1 yr (G3) *n* = 456	*χ*^2^ *p* value
Age in years (mean, s.d.)	25.9 (5.9)	25.4 (7.8)	32.1 (7.8)	
Marital Status (missing = 1, 0.1%)				<0.001
Married/Living as married	155 (70.5%)	37 (20.9%)	285 (62.5%)	
Single	62 (28.2%)	136 (76.8%)	140 (30.7%)	
Separated/Divorced/Widowed/Other	3 (1.4%)	4 (2.3%)	31 (6.8%)	
Living situation (missing = 3, 0.4%)				<0.001
Alone or with friends	1 (0.5%)	5 (2.8%)	10 (2.2%)	
Nuclear Family	167 (75.9%)	123 (69.5%)	347 (76.4%)	
Nuclear family + extended family	40 (18.2%)	27 (15.3%)	82 (18.1%)	
Extended family only	12 (5.5%)	22 (12.4)	15 (3.3%)	
Education (missing = 2, 0.2%)				0.097
Primary school or less	30 (13.7%)	14 (7.9%)	75 (16.4%)	
Some secondary/technical	121 (55.3%)	91 (51.4%)	225 (49.3%)	
Completed Secondary/technical	63 (28.8%)	67 (37.9%)	142 (31.1%)	
University or above	5 (2.3%)	5 (2.8%)	14 (3.1%)	
Occupation (missing = 3, 0.4%)				<0.001
Unemployed	124 (56.6%)	65 (36.9%)	168 (36.8%)	
Formal employment	45 (20.5%)	32 (18.2%)	171 (37.5%)	
Informal employment	21 (9.6%)	15 (8.5%)	82 (18.0%)	
Full-time student	29 (13.2%)	64 (36.4%)	35 (7.7%)	
Participant Type (missing = 1, 0.1%)				0.110
Patient	96 (43.6%)	95 (53.7%)	209 (45.8%)	
Non-patient	124 (56.4%)	82 (46.3%)	247 (54.2%)	
HIV Status (missing = 1, 0.1%)				<0.001
Positive	39 (17.7%)	37 (20.8%)	183 (40.1%)	
Other Chronic Illness (missing = 1, 0.1%)				<0.001
Yes	152 (69.1%)	106 (59.6%)	181 (39.7%)	

Table 2 displays the percentage of women in each group meeting criteria for CMDs based on responses to the MINI. Across the three subgroups, the prevalence of any disorder ranged from 35.3% to 40.7%. We did not find significant differences among the groups in terms of CMDs or suicide risk.

**Table 2.** *N* (and frequency) of psychiatric disorders among women (*N* = 853)

	Pregnant or child under one year (G1) *N* = 220	Women of childbearing age and without children (G2) *n* = 177	Woman with child older than 1 year (G3) *n* = 456	Totals	*χ*2 p-value
Any disorder	78 (35.5%)	72 (40.7%)	161 (35.3%)	311	0.43
Depression	71 (32.3%)	63 (35.6%)	142 (31.1%)	276	0.88
Anxiety	14 (6.4%)	10 (5.7%)	40 (8.8%)	64	0.31
Panic	5 (2.3%)	7 (4.0%)	15 (3.3%)	27	0.62
Suicidal ideation/risk	9 (4.1%)	16 (9.0%)	24 (5.3%)	49	0.09

Logistic regression models (Table 3) were used to compare the likelihood of a psychiatric disorder between groups, adjusting for sociodemographic and chronic illnesses covariates. No differences were detected among the different groups for the disorders assessed, except for two results related to suicide: Those women without children (G2), compared to pregnant/postpartum women (G1), had 2.61 times the odds of having a moderate/high suicide risk (95% CI 1.08–6.66), (*p* = 0.04) after controlling for demographic and clinical variables.

**Table 3.** Logistic Regression Models relating selected psychiatric outcomes to maternity status (*N* = 853)

	Depression	Suicide	Anxiety	Panic	Any disorder
Model 1					
G3 v. G1	0.95 (0.67–1.34)	1.30 (0.62–3.0)	1.41 (0.77–2.75)	1.46 (0.56–4.54)	0.99 (0.71–1.39)
G2 v. G1	1.16 (0.76–1.76)	2.33 (1.02–5.63)	0.88 (0.37–2.02)	1.77 (0.56–6.07)	1.25 (0.83–1.88)
G3 v. G2	0.82 (0.57–1.18)	0.56 (0.29–1.1)	1.61 (0.82–3.46)	0.83 (0.34–2.2)	0.80 (0.56–1.14)
Model 2					
G3 v. G1	1.04 (0.72–1.51)	1.44 (0.65–3.47)	1.61 (0.84–3.25)	1.67 (0.60–5.41)	1.07 (0.75–1.54)
G2 v. G1	1.06 (0.67–1.65)	2.95 (1.23–7.49)*	0.79 (0.32–1.91)	1.71 (0.48–6.5)	1.16 (0.75–1.8)
G3 v. G2	1.03 (0.69–1.54)	0.64 (0.32–1.32)	1.78 (0.85–4.06)	1.26 (0.47–3.72)	0.96 (0.65–1.42)
Model 3					
G3 v. G1	0.96 (0.66–1.4)	1.21 (0.54–2.94)	1.45 (0.75–2.94)	1.54 (0.55–4.98)	0.98 (0.68–1.42)
G2 v. G1	0.98 (0.62–1.55)	2.61 (1.08–6.66)*	0.71 (0.28–1.72)	1.65 (0.46–6.25)	1.09 (0.70–1.69)
G3 v. G2	0.98 (0.66–1.47)	0.47 (0.23–0.96)*	1.93 (0.92–4.37)	1.18 (0.43–3.49)	1.93 (0.92–4.37)

Model 1: Unadjusted.

Model 2: Age + marital status + occupation.

Model 3: Age + marital status + occupation + HIV status + presence of chronic illness.

*Note*: Bold values: *p < 0.05.

## Discussion

We found a high frequency of CMDs among women receiving care or accompanying patients in health care facilities in Mozambique, with about one-third of women meeting the criteria for major depression. These numbers are elevated compared with estimates of CMDs in high-income countries and other LMIC countries (Sawyer *et al*., 2010; Fisher *et al*., 2012), but consistent with studies in primary care conducted in South Africa (Stellenberg and Abrahams, 2015; Phukuta and Omole, 2020).

Contrary to our hypothesis, the frequency of CMDs did not vary based on maternity status (pregnant or mother of young child, mother of older children, and women without children). High rates of mental illness common to the three groups of our study can be, at least partially, explained by the fact that women of childbearing age in the Mozambican context are exposed to different types of socio-cultural pressures, elevating the risk of depression and other CMDs regardless of maternity status. These socio-cultural factors likely include gender inequality, the pressure to bear children, the high number of children to care for, high levels of HIV infection (as discussed below), low levels of education, low acceptance and inclusion of women in the workforce, and cultural expectations regarding women's role in society. Infertility, a lack of male children, unemployment, lack of education, submission to gender roles and duties, gender inequality, intimate partner violence, and social pressures are all factors that can contribute to high rates of common mental illness in Mozambican women in the three subgroups in such a way that no significant differences exist among the groups (Dyer, 2007; Lisboa and Letras, 2016). The lack of differences in frequency of psychiatric disorders by maternity status (pregnancy/perinatal v. mothers of older children v. women without children) is in line with the findings of Sawyer *et al*. (2010), in which researchers found similar rates of depression when comparing pregnant women and non-pregnant women. As described by Sawyer *et al*. (2010), however, there are several factors associated with pregnancy that may interfere with the identification of symptoms of depression. This supports the need for further research within the context of LMIC countries, so that we may better understand the characteristics of depression associated with differing maternity status, specifically in LMIC countries, where screening and treatment is already limited (Dos Santos *et al*., 2016).

Of note, these high rates of CMDs are seen in the context of a high burden of HIV infection and the presence of chronic diseases. Among our three study groups, the rate of positive HIV status ranges from 17.7% to 40.1%, which is in accordance with the elevated rates of HIV positive status in the country as a whole. In Mozambique, the data show that HIV affects women more than men, 15.4% and 10.1%, respectively, compared to the national rate which is 13.2% (Ministério Da Saúde- Misau and Instituto Nacional De Estatística – Ine, 2015). The stigma and discrimination surrounding HIV/AIDS can also contribute to the high rates of CMDs, as many women do not reveal their status for fear of the consequences they may face, particularly abandonment or violence (Ministério Da Saúde- Misau and Instituto Nacional De Estatística – Ine, 2015; Zhu *et al*., 2019).

Despite the lack of differences in CMDs by maternity status, we found that childless women of childbearing age had a higher likelihood of suicide risk than pregnant/postpartum women. The elevated rates in this specific group may reflect, as hypothesized, that childbearing is considered a central aspect of a woman's identity, with the absence of a child having a significant impact on a woman's life (Lisboa and Letras, 2016; Pinto, 2017) and possibly in her suicide risk. In addition, HIV infection, clearly elevated in this group, may play an essential role in the observed increase of suicide risk.

Although specifically elevated in childless women of childbearing age, suicide risk was high in the three groups, ranging between 4.1% and 9%. These are equivalent to rates described in some of the research literature, although higher than in some studies (Vijayakumar, 2015; Sweetland *et al*., 2019). Several existing studies find that factors related to pregnancy and the perinatal period that are common in Mozambique, such as young maternal age, unplanned pregnancy, poor support, or lack of a partner may contribute significantly to the higher rates of suicide risk during this phase of a woman's life (Patel *et al*., 1999; Orsolini *et al*., 2016). In addition, other social factors common to other stages of a woman's life, such as interpersonal violence, poverty, and lack of education, may also influence the risk of suicide (Sweetland *et al*., 2019).

Elevated rates of CMDs and suicide risk are correlated with poverty in a systematic review by Lund *et al*. (2010), in which 73–79% of the studies reported a positive relationship between various indicators of poverty and higher rates of poverty CMDs and risk for suicide. The pervasive degree of poverty in this context in Mozambique may explain the high percentage of CMDs and high suicide risk in this study.

Several limitations should be noted. Our study utilized a convenience sample of patients in primary care and thus cannot be interpreted as population prevalence. This sample may reflect an overestimation of population prevalence, as a woman with CMD may seek health care more often because of higher rates of comorbid physical illness or somatic presentation of mental health problems. Additionally, because participants were given a presentation addressing mental health and its definition, they may have been more likely to report having experienced certain symptoms, even though very few (1.4%) reported having been diagnosed with a mental illness. However, it is also possible that pregnant women with severe depression have more significant difficulties accessing these services and were not present during study recruitment.

Our study makes a unique contribution to the knowledge base on women's mental health by examining CMDs by maternity status in a low-income African country. Although CMDs were not significantly higher in pregnant/postpartum women compared to women who were not in the perinatal period in the present study, further studies in similar contexts addressing CMD in women in the perinatal period should be conducted, to bring additional evidence to support the improvement of mental health services for women in the perinatal period within these settings. Our finding that nearly a third of women of childbearing age in our sample meet criteria for CMDs regardless of maternity category suggests that mental health services are needed for all women of childbearing age. CMDs, besides causing suffering to the women themselves, also negatively impact their families and communities. Sustainable and scalable community-based stepped-care models of mental health services, such as those currently being evaluated for implementation in Mozambique (Wainberg *et al*., 2020, 2021), are poised to address this unmet need for mental health services for women of childbearing age.

## Conclusion

The frequency of CMDs among women of childbearing age in our study was high, especially compared to rates described in the literature in other low- and middle-income countries. We also found that for most disorders (except suicide risk), motherhood, despite all cultural expectations, was not a protective factor, and that pregnancy and the postnatal period were not necessarily a time of increased risk compared to women at other stages of the maternity process. Knowing this, healthcare providers might consider further exploring the integration of screening and evidence-based interventions that can be applied, even by those not specialized in mental health, as a way to reduce the treatment gap in pregnant women and all women of childbearing age.

## Data Availability

The datasets generated and/or analyzed during the current study are not publicly available due to regulations of the Mozambican government but are available from the corresponding author on reasonable request.
